# The effects of atorvastatin therapy on endothelıal function in patients with coronary artery disease

**DOI:** 10.1186/1476-7120-5-51

**Published:** 2007-12-30

**Authors:** Ahmet Yildiz, M Akif Cakar, Murat Baskurt, Barıs Okcun, Deniz Guzelsoy, Ugur Coskun

**Affiliations:** 1 Department of Cardiology, Gazi Hospital, İzmir, Turkey; 2 Department of Cardiology, Institute of Cardiology, İstanbul University, İstanbul, Turkey

## Abstract

**Background:**

Statins improve the endothelial function in patients with coronary artery disease (CAD). However, they contribute to the substantial decrease in coronary heart disease by reducing plasma cholesterol levels. They also, reduce oxidative stress, stabilize the atherosclerotic plaque and inhibit inflammatory response. These functions of statins have been briefly described as pleiotropic effects. The aim of our study was to evaluate the effect of atorvastatin therapy on endothelial functions in patients with CAD.

**Methods:**

Fourty-nine patients (40 men, 9 women, mean age 59 +/- 11 years) with diagnosed CAD were selected as the study group. The patients were given 10 mg/day atorvastatin for 12 weeks. If the target cholesterol levels has not been achieved 6 weeks after the treatment, then the daily atorvastatin dosage has been increased. The endothelial function was evaluated by flow mediated dilatation (FMD) of the brachial artery.

**Results:**

It has been figured out that 12 weeks later, atorvastatin caused a statistically significant decrease in the plasma levels of LDL-cholesterol and total cholesterol (p < 0,0001). Meanwhile, it was determined that the FMD got statistically significant improved 12 weeks after the atorvastatin therapy (8,1%–4,2%, p < 0,001). However there was no statistically significant change in non-endothelium dependent dilatation (NID).

**Conclusion:**

Endothelium derived vasodilatation (EBD), which was non-invasively detected via brachial artery ultrasonography, had statistically significant improvment within 12 weeks of atorvastatin therapy whereas non-endothelium dependent dilatation (NID) had no change.

## Introduction

The endothelium plays a vital role on the process of atherosclerosis; and it functions as a barrier between the blood and wall of the vessel [1,2]. Hypercholesterolemia is highly associated with impaired endothelial function. Endothelial dysfunction (ED) has a predictive value about the future cardiovascular events [3]. ED is reversible during the early stages of atherosclerosis. Some of the systemic markers of inflammation such as C-reactive protein (CRP) may also have predictive value for the future cardiovascular events in healty subjects, in elder patients and in individuals with high risk. The serum level of CRP is directly correlated with the presence and severity of coronary, cerebral and peripheral arterial atherosclerosis [4]. Beyond their lipid lowering effects, statins may improve endothelial function, reduce CRP and the risk of cardiovascular events. The endothelial function can be non-invasively evaluated by FMD of the brachial artery. The aim of our study was to evaluate the effects of atorvastatin therapy on serum lipid levels and to evaluate the effect of atorvastatin on endothelial function assessed by FMD of brachial artery in patients with diagnosed CAD.

## Materials and methods

### Study population

Fourty-nine patients (40 men, 9 women, mean age 59 +/- 11 years) with diagnosed CAD were selected as the study group. The inclusion cirteria for the study were described as; having coronary artery lesions on coronary angiography or previous myocardial infarction onset of which was older then 6 months, having a LDL-cholesterol level higher then 130 mg/dl and triglyceride level lower then 300 mg/dl and for diabetic population having a fasting glucose level lower then 180 mg/dl and HbA1c level below 8%. Our exclusion criteriae were; having a lipid lowering drug therapy for the last two months, undergoing any surgical operation within last 6 months, having an anticoagulant therapy, renal or hepatic failure, uncontrolled systemic hypertension (systolic>160 mmHg, diastolic > 100 mmHg), having a myocardial infarction, unstable angina, stroke, angioplasty and/or coronary artery bypass surgery within the period of last 6 months. Ten of 59 patients were excluded from the study due to the discordance in the treatment.

### Study design

All of the patients were evaluated at the begining of the study and at the 6th and 12th week of the study. Any patient who had not been taking aspirin therapy was given 300 mg aspirin daily and study was started two weeks later.

#### 1) Initial evaluation

At the beginning of the study a detailed medical history was obtained, demographic data including the heights and weights of the patients was collected and a detailed physical examination was performed. For analysing the fasting glucose level, lipid profile, liver functional tests, fibrinogen and CRP, blood samples were taken. For the assesment of the endothelial function before the treatment, brachial arterial ultrasonography which is a non-invasive test was performed. Later on all of the patients were given NCEP step 2 diet. Special attention was paid on not to change any other drug therapy that the patient has already been receiving. Atorvastatin dosage was ordered as 10 mg per day for patients whose initial LDL cholesterol levels were below 180 mg/dl and 20 mg per day for patients whose initial LDL levels were above 180 mg/dl.

#### 2) 6th week evaluation

The patients whose hepatic functional tests were two times higher then their initial levels were excluded from the study. The aim of the therapy was to achieve either a 30% decrease in the level of LDL cholesterol or a level under 125 mg/dl. When the expected level of LDL cholesterol was not reached then the atorvastatin dosage was increased to 20 mg per day for patients receiving 10 mg per day and to 30 mg per day for patients receiving 20 mg per day.

#### 3) 12th week evaluation

Blood samples were taken for the re-measurement of fasting glucose level, lipid profile, liver functional tests, fibrinogen and CRP and brachial arterial ultrasonography was repeated.

The vasomotor function of brachial artery can be evaluated by brachial arterial ultrasonography and this method is well correlated with coronary artery vasomotor function [5]. So, we preferred to perform brachial arterial ultrasonography for assessing the endothelial function. The protocol for the flow mediated dlation is performed according to guidelines, by Corretti. M.C. et al. [6] The medications such as nitrates, calcium antagonists, angiotensin converting enzyme inhibitors and beta blockers have been discontinued one day before the examination. The patients were wanted to lay down in supine position. Two-dimensional images of 10–20 mm slices of the right brachial artery were obtained via 8,5 MHz linear transducer. Later on, the largest diameter of the artery was measured during systole via M-mode imaging. The measurements were repeated three times and the arithmetic medians were calculated. After the measurement, 250 mmHg pressure was applied to the same upper extremity with a cuff for 5 minutes. After loosening the cuff, the ultrasonographic views were recorded for 3 minutes and the largest diameter of the vessel in systole was measured (Endothelium dependent vasodilatation-EDV) (Reactive hyperemia). After the diameter of the vessels returned to their resting state (10 minutes later), the patient was given 2,5 mg sublingual nitroglyicerin spray and then the images were recorded for 5 minutes and the largest diameter of the vessel during systole was reached (Vasodilatation independent from endothelium-NID).

Rates of EDV and NID were obtained in percentage according to the ratio between the arterial diameters measured at rest and arterial diameters measured after reactive hyperemia and application of nitroglyicerin. The calculation was made according to the formula below.

% change = (D2-D1)/D1 × 100%

D1 was the initial diameter while D2 was the diameter measured after atorvastatin therapy.

### Plasma lipid and C-Reactive Protein levels

Lipid profiles and hepatic functional tests of the patients were analyzed at the beginning, at the sixth week and at third month of the therapy. The total cholesterol levels were measured by Technicon Opera autoanalyser by using trading kit (biotrol) after it dissolved. CRP was checked by using high sensitive Dade Behring kit on Dade Behring Nephhelometer 100 device. Dilution was applied to high levels. CRP levels under 0,34 mg/L were accepted as normal while the levels higher were accepted as pathological.

### Statistical analysis

In our study, quantitive factors were indicated as aritmetic means +/- standard deviation and qualitative factors were indicated as percentage. Matched t test was used for the comparison of the brachial arterial diameters. The total cholesterol level was evaluated via Pearson correlation analysis according to the % change between LDL and EBD. The correlation was considered as statistically significant for whom the coefficient of correlation was higher then 0,05. The SPSS 11,0 package statistics program was used for statistical work.

## Results

The demographical characteristics, coronary angiographic findings and the medications have been summarised in Table 1. Initially, 10 mg atorvastatin was given to 41 patients (83,6%) and 20 mg to 8 patients (16,3%) and the dosages were increased substantially according to the control levels at the sixth week of the therapy. No significant increase was seen in the liver functional tests in any control evaluation.

**Table 1:** The demographical and angiographic characteristics of patients

Age (year)	59. 33 ± 11. 20
Sex	
(men, n=)	40 (% 81. 6)
(women, n=)	9 (% 18. 4)
BMI(kg/m^2^)	27. 50 ± 3. 41
Hypertension	23 (%. 47)
Diabetes	12 (% 24. 5)
Smoking	18 (% 36. 7)
Family history	13 (% 26. 5)
The diameter of Brachial artery	
(men)	3.87 ± 0.41
(women)	3.56 ± 0.41
EF %	48. 39 ± 7. 37
Previous MI	33/49
Coronary angiography.	44/49
One vessel	13/49
Multivessel	32/49
Previous revascularization (CABG or PCI)	33/49

EF:Ejection fraction

### Effects of atorvastatin on serum lipid and CRP levels

It has been seen that the total cholesterol and LDL cholesterol levels got significantly decreased at the third month of the atorvastatin therapy. The total decrease was 32,4% for total cholesterol level and was 42,3% for LDL cholesterol level. The triglyceride levels and HDL cholesterol levels were not changed significantly. With atorvastatin treatment, the rate of decreament in fibrinogen levels was 8,2 % (p < 0,05) while it was 31,6 % (p < 0,001) in CRP levels.

### The results of brachial arterial ultrasonography

The brachial artery diameter was 3,81 +/- 0,43 mm at resting state before treatment. At the third month of the atorvastatin therapy, the new resting diameter was 3,82 +/- 0,43 mm and the change had no statistically significant meaning (p > 0,05). The dilatation formed in brachial artery during hyperemia caused by cuff pressure (EDV) was measured as 4,25 +/- 1,7 % at the beginning of the therapy and 8,16 +/- 1,84 after 3 months of atorvastatin therapy. The EDV values were increased significantly after three months of the therapy (p < 0,0001). When compared with the beginning of the therapy, it has been seen that after application of 2,5 mg sublingual nitroglycerin the vasodilatation independent from endothelium (NID) did not get changed significantly after 3 months (8,74 +/- 2,58% vs 8,73 +/- 2,34, p > 0,05).

## Discussion

In recent years the trials mainly have focused on non-invasive detection of endothelial dysfunction which has been accepted as an early sign of atherosclerosis. This non-invasive method is based on determination of EDV and NID via high resolution ultrasonography of brachial artery [7,8].

There are several agents which affect endothelial functions. Among these agents, Angiotensin Converting Enzyme (ACE) inhibitors are the most widely known agents bearing such an endothelial activity [9,10].

It has been figured out that hypercholesterolemia causes no impairment on endothelial functions in premenopausal women. This has been considered to be due to the positive effects of estrogen on endothelial functions. It has been reported that the endothelial functions could be improved by the use of aspirin in athersclerotic patients and this has been considered to be associated with the inhibition of constructor substance formation by aspirin via the cyclooxygenase pathway [11].

Hypercholesterolemia is the most important risk factor in the development of coronary atherosclerosis [12]. The deterioration of endothelium dependent on vasodilatation has been found to be related with hypercholesterolemia [12-15].

The oxidized LDL, which gets increased in hypercholesterolemia and atherosclerosis cause impairment of endothelial vasodilatation by decreasing NO synthase activity [16]. The deterioration of EBD takes place before the formation of atherosclerotic lesions [12-14]. Hypercholesterolemia causes deteriation of EBD and then myocardial perfusion decreases [12-17]. Statins enhance the endothelial vasodilator and fibrinoliytic capacity by lowering the oxidized LDL.

The use of statins in patients with CAD and hyperlipidemia results in improvement of both endothelial functions of coronary arteries and myocardial perfusion [18-21]. The main causative substrate for this improvement is endothelium derived relaxing factor (EDRF = NO). Within 24 weeks of fluvastatin therapy, an increase in the forearm blood flow through plethysmography has been detected in hyperlipidemic patients with an inverse ratio of LDL levels [22]. The same benefical effect has been also reported with 4 weeks of simvastatin therapy [23]. In RECIFE study, improvement in endothelial functions with pravastatin use (40 mg/d) has been shown in such a short time as 6 weeks [24].

In a study, Anderson et al. randomized 49 patients into three groups with average serum cholesterol levels of 209 +/- 33 mg/dl [25]. At the end of the first year, a significant improvement in the coronary artery dilatation by Ach has been observed. Consequently, the authors indicated that an improvement in endothelial functions could be obtained in patients with CAD and unfavorable cardiac events could be reduced with the combination of cholesterol lowering therapy with antioxidant therapy.

In our study we hold a group that contained patients with high risk (diagnosed CAD, 30% incidence of diabetes mellitus) and we examined the effect of atorvastatin on endothelial functions, by evaluating the EDV and NID on brachial artery before and after 12 weeks of atorvastatin therapy. At the end of 12 weeks, LDL cholesterol levels showed a decrease of 42,3 % and endothelium dependent vasodilatation showed a 92% increase. We found no significant change in NID with atorvastatin therapy. Our results were similar to the other statin studies (RECIFE etc.) [24]. However. the method we used is a very simple method highlighting the general status of the circulatory system. Because it depends on ultrasonography, the examination requires experience.

As a result, in the adults who have normal vascular function, a marked improvement in endothelial functions with 80 mg/day atorvastatin therapy was observed within 24 hours and this improvement came out before cholesterol lowering activity. However, it has been seen that vascular functions deteriorated acutely irrespective of cholesterol levels after cessation of atorvastatin therapy [26].

## Conclusion

Statins have cholesterol lowering effects and recover endothelial dysfunction which contributes to atherosclerosis in patients with hypercholesterolemic ischemic cardiac disease. The improvement in endothelial functions may play a significant role in reducing the risk of future coronary events. It is important to use statins for a long time, because of rapid deterioration of endothelial functions occurs shortly after cessation of statin therapy.
